# Language: Its Origin and Ongoing Evolution

**DOI:** 10.3390/jintelligence11040061

**Published:** 2023-03-28

**Authors:** Ilia Markov, Kseniia Kharitonova, Elena L. Grigorenko

**Affiliations:** 1 Department of Psychology, University of Houston, Houston, TX 77204, USA; 2 Texas Institute for Measurement, Evaluation, and Statistics (TIMES), The University of Houston, Houston, TX 77204, USA; 3 Center for Cognitive Sciences, Sirius University for Science and Technology, Sochi 354340, Russia; 4 Baylor College of Medicine, Houston, TX 77030, USA; 5 Child Study Center and Haskins Laboratories, Yale University, New Haven, CT 06520, USA; 6 Rector’s Office, Moscow State University for Psychology and Education, Moscow 127051, Russia

**Keywords:** communication, evolution, language

## Abstract

With the present paper, we sought to use research findings to illustrate the following thesis: the evolution of language follows the principles of human evolution. We argued that language does not exist for its own sake, it is one of a multitude of skills that developed to achieve a shared communicative goal, and all its features are reflective of this. Ongoing emerging language adaptations strive to better fit the present state of the human species. Theories of language have evolved from a single-modality to multimodal, from human-specific to usage-based and goal-driven. We proposed that language should be viewed as a multitude of communication techniques that have developed and are developing in response to selective pressure. The precise nature of language is shaped by the needs of the species (arguably, uniquely *H. sapiens*) utilizing it, and the emergence of new situational adaptations, as well as new forms and types of human language, demonstrates that language includes an act driven by a communicative goal. This article serves as an overview of the current state of psycholinguistic research on the topic of language evolution.

## 1. Introduction

Research on language origin and evolution may be viewed as two of the most prominent research directions of the past few years. While these questions have been at the forefront of language science since its inception, only recently have we seen methodologies and techniques being developed that can provide answers backed with sufficient empirical evidence. The landscape of theoretical frameworks of language origin, the form in which it originated, and its worldwide dispersal has also been shifting in response to newly obtained evidence. The field of language evolution research can be described as currently coming of age while already equipped with a rich toolkit of methods for pursuits such as comparative research, investigating commonalities and differences between human language and animal communication systems, and studying cumulative cultural evolution of communication systems in experimental settings (Dediu and de Boer 2016).

The aim of this article was to give a brief overview of the evolution of language, as well as to demonstrate how theoretical frameworks of language origin and evolution evolved with it. We sought to utilize the latest findings to argue that evolution is one of the central driving forces of the existence and development of human language. Language is a human skill, the nature and features of which are shaped in accordance with the needs of the species through continuous usage and adherence to communicative goals (Grigorenko 2023). That nature is reflected through newly emergent language origin theories that move away from the innateness of language and provide plausible explanations of gradual language emergence from a multitude of other subsystems of communication. In regard to language modality, the main informational channel of origin, we intended to provide theoretical reasoning and evidence for the multimodal approach.

To illustrate that language is a skill that constantly undergoes changes due to various selective pressures, we aimed to explore three groups of factors that seem to transform language in the most significant ways: factors of the physical environment (such as aridity, vegetation, ambient temperatures, precipitation, latitude), socio-demographic factors (number of language users, geographic spread, degree of language contact, and the role of communicative situations), and technological advances (Internet, smartphones, and instant messaging). The latter group of factors is of most interest to us due to the changes that online communication is bringing about at a rate that has never been witnessed before. We propose that the evolution of language follows a similar pattern to the one outlined by recent research into general intelligence, promoting a more context-dependent and dynamic view of intelligence that is focused more radically than ever before on niche construction as a result of the cultural evolution (Preiss 2022). The factors of the environment that influence language now reflect the changes brought along with the Anthropocene (Anthropocene Working Group 2019), which further alters the ecological niche of our species, necessitating further adaptation.

While the argumentation provided in the present article is split into two main sections, covering the theories of origin and the evidence of evolution, all of it follows the main thesis of language emerging and evolving through usage; we deem this thesis to be the most prominent new direction in language research.

## 2. Origin of Language

There are more than 7000 living languages across the globe today (Lewis 2009). To approach the topic of the ongoing evolution of language that spans millennia, we first need to determine its origin. However, the question by itself poses a challenge.

An important philosophical distinction collects two separate topics under the label “origin of language”: the origin of language faculty and the origin of languages (Formigari 2013). The latter leads to a further question, namely, whether all world languages derive from one single “protolanguage”—i.e., monogenesis—or do several language families (e.g., Indo-European, Afro-Asiatic, Altaic) each derive from a different protolanguage—i.e., polygenesis (Graffi 2019).

The theories relating to the emergence of language have been extensively debated for centuries, once leading to the infamous ban of the germane discussions by the Société de Linguistique de Paris in 1861. However, the field has seen a surge in this theorizing since the late 20th century and its considerable evolution over the past two decades (Nölle et al. 2020b). The focus of the debate has been on the innateness of language for many years. The central line of research that incorporates the traditional generative view (Chomsky 1988), notions of innateness, universal grammar (UG), and the poverty of stimulus argument is largely classified as biolinguistics (for a more modern iteration of this framework, see (Boeckx 2021; Bolhuis et al. 2014; Hauser et al. 2002)). Such approaches argue that knowledge of language structures is impossible to extract from linguistic input; hence, it is suggested to be innate. An opposing point of view, referred to as “usage-based” or “emergentist” approaches, postulates that language emerged from its usage: “meaning is use—structure emerges from use” (Tomasello 2009, p. 69); linguistic knowledge in these approaches proceeds via the abstraction and schematization of actual language use into fixed chunks, as well as more abstract linguistic patterns that become cognitively entrenched. These approaches, therefore, reject the notion of UG (Pleyer and Hartmann 2019), instead utilizing the notion of the common communicative goal to explain language commonalities (Arbib 2012). Notably, attempts to synthesize the opposing theories were few and far between. Such an attempt was undertaken by Pinker and Bloom (1990), where the authors put forth a compelling case against viewing language as a “spandrel”—an architectural allegory of a space formed at the intersection of other spaces, its shape therefore not being a significant trait on its own—arguing that certain constraints would not allow us to assume a non-adaptationist point of view, as “no adaptive organ can be adaptive in every aspect” (1990, p. 19).

In these controversies, one of the most contested topics is the question of causality, which is influenced by several factors: the problem of spurious correlation (Roberts and Winters 2012), the universally concerning “replication crisis” (Open Science Collaboration 2015), and a tendency to rely on indirect evidence (Nölle et al. 2020b). A novel solution for this contest is termed the “maximum robustness approach,” which, instead of focusing on simple causal relationships, aims to systematically construct more complex and coherent causal graphs (Pearl 2000), incorporating all available evidence to form links between multiple variables. The CHIELD database, which is a repository of linguistic hypotheses produced in literature, was created to explore such graphs in order to find gaps or conflicting relationships, which can help to design empirical research addressing these issues and uncovering actual causal mechanisms (Roberts et al. 2020). The database is public and functional: it is designed to be extendable by future researchers to ultimately become comprehensive and inclusive of as many languages as possible (that exist). Universal acceptance of a database such as this is the first step toward a realistic implementation of the maximum robustness approach.

To summarize, the current general trend for the linguistic field seems to move further and further away from notions of language innateness, although significant support for these viewpoints remains. In the scope of language evolution, usage-based frameworks allow for significantly more detailed and insightful investigations into the emergence of language. The same appears to be true for research into the modality of origin: this landscape of theories is also changing.

### 2.1. Modality of Origin

The debate on the modality of origin, which is the initial main channel carrying verbal information, also has a few contesting theories: according to the “gesture-first” view, “language evolved initially from manual gestures with vocal elements gradually added” (Corballis 2011, p. 383). The “speech-first” view (Dunbar 1997; MacNeilage 2008) argues for the pre-emergence of a vocal–auditory modality given its present-day dominance (for a full historical overview, see Fitch 2010). Modern theories argue for a multimodal emergence theory, incorporating complex interplay between auditory and visual channels (Perlman 2017). Among newer ideas, “pantomime-first” was put forward as a distinct theoretical proposal (Zlatev et al. 2017), which intrigues but does not provide much empirical evidence for its support. Another supporting usage-based account on multimodality comes from Levinson and Holler (2014), who propose that language normally occurs while embedded in a layered structure of multiple other channels of information. This view enables different phylogenetic and evolutionary origins to be assigned to each layer. Such holistic representation helps to bridge the gulf between the species, allowing us to recognize precursor adaptations such as turn-taking in current primates and the gestural skills of great apes as the first steps toward language formation, while the whole ensemble of language continues to be distinctively human.

One of the novel multimodal hypotheses is the mirror system hypothesis developed by Rizzolatti and Arbib (1998), which postulates that the mechanisms that support language in the human brain evolved atop a basic mechanism not originally related to communication: the mirror system, as the evolutionary basis for language, possesses a capacity to generate and recognize a set of actions. Arbib argues that the evolution of language is rooted in the execution and observation of hand movements, leading to the emergence of sign language, which was thereafter extended to speech. Complex imitation for hand movements evolved adaptively because of its utility in the social sharing of practical and manual skills. Skill sharing through imitation, such as grasping objects and using simple tools, existed long before language, being “more powerful than the call and gesture systems of nonhuman primates but lacking the full richness of modern human languages” (Arbib 2012, p. 157).

Importantly, the origin theories based on the writing modality are characteristically absent, which is understandable given its (mostly) secondary nature to spoken language. This, however, is all too indicative of the attitudes to writing in language research prior to modern studies. The linguistic views on the emergence of writing were varied and controversial, echoing many general issues of the evolution of spoken language. The traditional outlook on writing systems since Aristotle was superficial, ostensibly viewing these systems as an optional, supplemental representation of spoken language. Moreover, writing was deemed a “wandering outcast of linguistics” (Derrida 1976), leading to a suppression of research on writing. Similarly, for Saussure, written language was an object of suspicion, presenting a confounding and contaminating influence on language, going so far as to state that “to let go of the letter means a first step in the direction of truth” (Saussure et al. 1986, p. 32). The views that were expressed during that time in the field were later ascribed to the “written language bias” (Linell 2004).

Views that contested that bias started emerging in the mid-20th century from the historical (Goody 1986) and anthropological (McLuhan 1962) fields that, in turn, influenced studies of language to ascribe a more fundamental meaning to writing. One of the modern points of view from D.S. Olson proposed a special relationship of writing to the general machinery of language, which was influenced by those accounts and driven by developmental evidence (Robinson et al. 1983). Olson’s most recent account postulated that reading and writing create a system of meta-representation concepts that contribute to consciousness, the formation of systematic thought, and rationality. In addition, some theories suggest that writing did not emerge as a secondary representation of spoken language but as the evolution of the token system for the purposes of goods exchanged or accounting (Schmandt-Besserat 2012). The role of writing systems is similarly far from secondary according to the literacy hypothesis (Goody and Watt 1963), and while it has received a lot of criticism on the matter of most aspects of civilized society preexisting and assimilating literacy at its advent, some scholars define influential “biases” (Olson 2012) that may have contributed greatly to the cognitive and social development of the species. Such an impact of writing may be evidenced by tests of intelligence, including items that deal with vocabulary and the relationship between words, which test our capacity to participate in a literate environment (Olson 2005, as cited in Preiss and Sternberg 2006). Additionally, writing is essential to consider if adopting the adaptationist point of view, as the emergence of writing seems to possess several features characteristic of a Darwinian process (Lock and Gers 2012).

Thus, while the dominating role of the vocal–auditory modality remains indisputable, progress in the field was made toward developing multimodal theories of language origin, which aid in unifying disparate evidence in support of different single-modality theories under a single governing principle.

### 2.2. Origin of Languages

The second question out of the pair laid out at the beginning of the section, namely, regarding “the origin of languages,” was mostly inquired upon in neighboring fields of inquiry and tied to the spread of human populations. A link between the human genome and the spread of languages has been debated ever since Darwin proposed that “a perfect pedigree of mankind… would afford the best classification of the various languages now spoken throughout the world” (Darwin 1871). While some argued that the spread of languages is a good proxy for the dispersion of human populations (Gray and Atkinson 2003; Mace and Holden 2005), opposition to this assumption was also persistent (e.g., Donohue and Denham 2010). Quantitative evidence supports both a general gene–language dispersion correspondence but also substantial (~20%) mismatches between 10 language families and corresponding populations (Barbieri et al. 2022).

Globally, a consensus around the serial founder effect (SFE) process playing an important role in shaping global patterns of neutral genetic diversity is currently forming. This process entails a series of population splits, movements into an unoccupied territory, and subsequent isolation: beginning in Africa and proceeding through Eurasia into the Americas and Oceania. At the within-population level, it led to a steady decay in genetic diversity with increasing geographic distance from East Africa; at the between-population level, it led to a steady increase in genetic distance with increasing geographic distance (Prugnolle et al. 2005; Ramachandran et al. 2005). The debate on the topic of language dispersion was later reignited by Atkinson (2011), who proposed that phoneme inventories in human languages had undergone a parallel SFE process based on the finding that the number of phonemes in 504 widespread languages decreased linearly with increasing geographic distance from Africa. Alternative assumptions for worldwide phonemic cline were tested using numerical simulations, showing that this pattern may be due to a repeated bottleneck effect and phonemic loss: low-density populations lost phonemes during the out-of-Africa dispersal of modern humans (Pérez-Losada and Fort 2018). Creanza et al. (2015) further delved into this issue by performing joint and parallel analyses of phoneme counts in 2,082 languages and DNA microsatellite polymorphisms, which were used as signatures of human demographic history to calculate genetic distances between 246 populations. The results decisively vindicated Darwin’s proposal of human races and languages evolving in concert following a tree-like history of splits and isolation (Darwin 1860) at the global level; however, it did not align with the SFE model with Africa as the center of origin, with it instead being more inclined toward a Eurasian-centered model. A more recent and novel analysis, which covered a cultural layer adjacent to language, namely, music, was carried out on a dataset of 152 societies (containing 1,054 songs from the public database The Global Jukebox in the form of raw coded Cantometrics data, 1,719 genomic profiles, and 152 languages); the analysis demonstrated weak links between music and language (R^2^ <= 0.05), as well as with genetic distance and geographic proximity, in contrast to the much stronger relationships found between genes and geography: the results suggest that genes and culture are surprisingly decoupled (Passmore et al. 2022). For the Indo-European family, Bouckaert et al. (2012) used Bayesian phylogeographic approaches with a dataset of basic vocabulary term lists from 103 ancient and contemporary Indo-European languages to model the expansion of the family, finding decisive support for an Anatolian origin over a steppe origin, with both the inferred timing and root location of the Indo-European language trees fitting with an agricultural expansion from Anatolia beginning 8000 to 9500 years ago. Certain linguistic methods, such as Bayesian phylogeographic approaches, that emerged from recent studies provided tentative answers to general questions of human prehistory: a recent study using lexical data and Bayesian phylogenetic methods placed the Austronesian origin in Taiwan approximately 5230 years ago and supported the hypothesis of “pulse-pause” expansion from Taiwan on the origin of the Austronesian settlers of the Pacific (Gray et al. 2009). While being fairly recent for the field, the aforementioned techniques succeeded in helping linguists reclaim the issue of the origin of language as the viable research aim for future investigations aside from the origin of humanity research in neighboring fields.

To summarize, new technological and methodological advances have led to the most drastic changes in language evolution research. Large-scale investigations do require substantial resources, and interdisciplinary collaboration poses a challenge, but the results obtained contribute to significant advancements in the linguistic and neighboring fields in regard to the origin of language dispersal.

### 2.3. Neural Correlates of Language

The final important aspect of language origin studies, tangential to linguistics but central to psycholinguistics, is of utmost importance for the present essay: neural correlates of language. Evidence obtained from numerous previous studies that attempted to localize language within the brain is well established: the clinical studies of Broca (Broca 1861) in the 19th century and Wernicke (Wernicke [1874] 1994) in the 20th century, although contested now, served as an initial impulse for this. Similar to the debate on the language faculty origin in linguistic circles, we can note that the initial research findings that focused on narrow specificity of function were later extended to cover contesting evidence and new theoretical frameworks and have evolved into a multidimensional system. Through further publications on the topic emphasizing the importance of previously unaccounted-for brain regions (e.g., insula, Dronkers 1996), the model of choice for the end of the 20th century became the aphasia model (e.g., Obler and Gjerlow 1999). At the beginning of the 21st century, the model was further expanded into Broca-type and Wernicke-type aphasias in accordance with the impairment in one of two language comprehension axes (Ardila 2011, 2012), further solidifying the trend toward system complexity.

Modern models of language include numerous areas of the brain organized in multiple circuits within clusters of activation (Ardila et al. 2016). One such model was constructed by Peter Hagoort (2005), who argued that the operation of distributed neural networks in Broca’s area and the left inferior frontal gyrus (LIFG) involves parallel processing of semantic, syntactic, and phonological information through three functional components: memory (long-term memory retrieval), unification (integrating information), and control (selecting a language “action”). Evidence from EEG and MEG studies helped to identify the specific temporal features of unification and memory retrieval components, arguing for neuronal synchronization that supports functional interrelatedness rather than strict domain specificity (Bastiaansen and Hagoort 2006). These considerations are far from a theoretical conjecture nowadays, as they have been translated into presurgical planning (Alemi et al. 2018). Additionally, there is evidence of neural multifunctionality for language networks, in particular, several frontal networks being linked to non-linguistic functions, such as mental rotation (Jordan et al. 2001), musical syntax processing (Maess et al. 2001), and arithmetic comprehension (Baldo and Dronkers 2007). Such findings have driven researchers toward frameworks of multifunctional modularity and are instrumental for scholars developing usage-based approaches to language evolution.

Thus, despite initial findings that focused on the narrow specificity of function, the field has evolved to include numerous areas of the brain organized in multiple circuits within clusters of activation, in addition to the emerging evidence of neural multifunctionality for language networks.

## 3. Language Adaptation

Lupyan and Dale (2016) argued that observed linguistic differences arise not only from the accumulation of random changes due to the languages drifting apart but also may be reflective of the environment in which the language was developing. These environmental aspects that pressure languages into continuous diversification are social, physical, and technological in nature (Lupyan and Dale 2016).

Just like birds develop different beaks adapting to different environments, languages and cultures might be undergoing similar changes (Lupyan and Dale 2016). Charles Darwin, in “The Descent of Man, and Selection in Relation to Sex,” cited Max Müller to make a case for the evolution of language: “A struggle for life is constantly going on amongst the words and grammatical forms in each language. The better, the shorter, the easier forms are constantly gaining the upper hand, and they owe their success to their own inherent virtue.” (Darwin 1871, p. 58). However, this idea of progress in linguistic evolution is considered dysfunctional by some (Labov 1991; Mendívil-Giró 2018) due to its inability to explain the main patterns of linguistic structural diversity; a growing body of research asserts the contrary. The process of language diversification cannot be understood without considering the pressures that several factors (physical, ecological, and social) put on language users in different environments (Bentz et al. 2018).

### 3.1. Ecological Adaptations

Similar to the communication systems of other species, language may be affected by ecological factors. Physiologically based predictions demonstrate that languages with complex tonality have generally not developed in very cold or otherwise desiccated climates, as air dryness decreases the control of the vocal folds and pitch production, and this, in turn, results in the absence of a (complex) tone system. The geographic–linguistic association operates within continents, major language families, and across language isolates (Everett et al. 2015). However, replication of the study on a different dataset found it was not robust (Roberts 2018). An analysis of over 4,000 language varieties showed a positive association between the language’s degree of reliance on vowels and the typical ambient humidity of a language’s native locale, which is consistent with other studies that focus on the link between aridity (i.e., the lack of effective moisture in a climate) and tonality of language (Everett et al. 2015; Everett 2017), but the robustness was later found to be limited (Roberts 2018).

Environments in which higher sound frequencies are less faithfully transmitted due to denser vegetation or higher ambient temperatures seem to be related to the greater use of sounds of lower frequencies (“more sonorous” languages). The results of Maddieson and Coupé (2015) point to a significant relationship between the “consonant-heaviness” of languages and several environmental factors, including tree cover and precipitation (Maddieson and Coupé 2015). Further analysis of spoken samples did not find the relationship significant but identified that the percentage of sonorous material is correlated with the mean annual temperature in the area of the language (Maddieson 2018). Studies that focused on the influence of temperature on languages find that languages spoken in cold, small regions tend to be more complex across a range of linguistic features, such as morphosyntactic complexity, linguistic diversity, word length, and consonant inventory (Lewis and Frank 2016).

Another striking example is the observed partial correlation between latitude and the absence or presence of the word for the color blue (Brown and Lindsey 2004; Lindsey and Brown 2002) due to the negative impact of ultraviolet light (UV-B) on the perception of the blue/green distinction (phototoxicity). In high-UV areas, languages without the word for blue prevail, which also correlates with the rates of blue-yellow color vision deficiency in these areas suggesting an evolutionary, physiological cause for both phenomena (Brown and Lindsey 2004; Dediu et al. 2017).

A common criticism of the abovementioned studies is that they are correlational in nature, thus, do not contribute to the understanding of possible mechanisms that underlie linguistic evolutionary processes. In order to improve the methodological robustness of the studies, additional approaches, such as iterated learning, a historical case study, corpus studies, and studying individual speech, was suggested (Roberts 2018). For this reason, several studies tried to experimentally investigate how environmental factors drive the emergence of linguistic conventions. Nölle et al. (2020a) adapted the classical maze game task to confirm that subtle environmental motivations cause the emergence of different communicative conventions in an otherwise identical task, pointing to linguistic adaptations being highly sensitive to factors of the shared task environment. The authors speculated that these kinds of mechanisms identified at a local interactional level might contribute to the systematic global variation observed between different languages.

One of the most striking examples of linguistic adaptation to the environment is whistled languages. The main purpose of whistled languages is to facilitate spoken communication at great distances, but it is also used in other circumstances, such as secrecy, courtship, singing, and communication in noisy environments. Although they are always referred to as languages, they are considered a mode of speech because whistled languages are always based on a spoken language (Meyer 2015).

Several hypotheses were put forward to explain the current existence of whistled languages. One of them posits that whistled languages are simply the vestigial remains of a widespread ancient phenomenon. This mode of speech could have been used by prehistoric hunter-gatherers for hunting in groups or signaling a danger in any type of environment (Nettle and Romaine 2000). Another possible explanation is that the actual whistled languages are found only in a small minority of languages due to the erosion of traditional lifestyles and the relative ease of resorting to shouting because whistled speech would generally require more pressure to develop. This argument would be in favor of a key role played by significant environmental constraints in the emergence of whistled speech, which is supported by the observed systematic adaptation of whistled speech to typically constraining and geographically scattered ecological milieux (Meyer 2015).

It is estimated that approximately 70–80 languages actively use their whistled mode of speech, but the number is rapidly declining due to modern technologies of communication, and most of them are endangered (Meyer 2018). Evidently, whistled speech plays a strong functional role by complementing regular speech under unusual circumstances. Around the world, whistled forms of languages are associated with traditional activities, such as hunting, hill agriculture, or shepherding, in which individuals are relatively isolated and scattered across substantial areas of densely vegetated landscapes. In this type of environment, whistling has a clear advantage over speaking or shouting: acoustic signals can easily overcome ambient conditions and can travel longer distances. For example, La Gomera, one of Spain’s Canary Islands, holds the record for the longest distance of whistled conversations of approximately 1 km (Meyer 2015); others have observed communications at approximately 8 km (Busnel and Classe 1976).

The principle of whistled speech is straightforward: people articulate words while whistling, which involves acoustic reduction at the produced frequency level and selection of key salient phonetic cues for the corresponding spoken utterances. The resulting signal’s linguistic structure is identical to standard speech. Interestingly, even though the acoustic channel is reduced, whistled sentences remain highly intelligible to trained speakers (Meyer 2015).

It was suggested that human whistled languages can serve as a model for understanding the coding of information in dolphin whistle communication. Comparing human and dolphin whistles could become a complementary test bench for the development of new methodologies for decoding whistled communication signals by providing new perspectives on structural and organizational aspects of encoding information (Meyer et al. 2021).

Overall, exploring the connection between the structure of languages and the environment in which they are utilized is complicated by several issues. If the ecology of the area was able to influence the language at the stage of its emergence, the amount of information needed to make a conclusive statement about it is scarce. Additionally, most of the studies that focus on the link between environment and language are correlational in nature. Although the overall structural diversity of languages has not been linked with other types of diversity, some aspects, such as morphosyntactic complexity or consonant/vowel inventory, may be affected.

### 3.2. Socio-Demographic Adaptations

Apart from the effects of the physical environment and location, languages may be shaped by social and demographic factors. A statistical analysis of 2000 languages revealed strong relationships between the morphological complexity of a language and demographic/socio-historical factors, including the number of language users, geographic spread, and degree of language contact (Lupyan and Dale 2010). It was suggested that languages spoken by large groups have a simpler inflectional morphology than languages spoken by smaller groups. Additionally, languages spoken by large groups are more likely to utilize lexical strategies in place of inflectional morphology when encoding evidentiality, negation, aspect, and possession (Lupyan and Dale 2010). Based on these findings, Dale and Lupyan proposed the *linguistic niche hypothesis*, which describes the esoteric and exoteric niches for languages. The exoteric linguistic niche includes languages with large numbers of speakers (e.g., English, Swahili, and Hindi), which forces these languages to serve as a means of communication between strangers. Speakers of languages in the exoteric niche, compared with the esoteric niche, are more likely to be non-native speakers or have learned the language from non-native speakers and use the language to speak to individuals from different ethnic and/or linguistic backgrounds. The esoteric niche includes languages like Tatar, Elfdalian, and Algonquin (Dale and Lupyan 2012; Lupyan and Dale 2010, 2016). Linguistically, esoteric languages are more likely to be classified as isolating rather than fusional, have fewer grammatical categories marked on the verb, are more likely to encode negation via analytical strategies than using inflections, are less likely to have indefinite and definite articles, and are less likely to communicate distance distinction demonstratives (Lupyan and Dale 2010). Further studies found only limited support for this hypothesis (Lewis and Frank 2016) or did not find a strong relationship between the grammatical or statistical structure of language and the proportion of non-native speakers (Koplenig 2019).

Winters et al. (2015) experimentally investigated the role of the communicative situation in which an utterance is produced and how it influences the emergence of three types of linguistic systems: underspecified languages, holistic systems, and systematic languages. Using a discrimination task in a communication game and manipulating whether the feature dimension shape was relevant or not in discriminating between two referents, it was established that different linguistic systems emerged. Furthermore, experimental languages gradually developed to encode information relevant to the communicative task in a given situational context. These results suggest that language systems adapt to their contextual niche over iterated learning.

Another interesting observation was made about the influence of population size on rates of language evolution. The rates of gain and loss of cognate words for basic vocabulary were analyzed in Polynesian languages. Larger populations were observed to have higher rates of gain of new words, while smaller populations had higher rates of word loss, which suggests that demographic factors may affect rates of language evolution and that rates of gain and loss are affected in different ways. However, the authors found that the results were strikingly consistent with general predictions of evolutionary models, paralleling positive selection in the case of greater rates of word gain in larger populations, and loss of diversity in small populations and greater rates of word loss (Bromham et al. 2015).

An inquiry into language evolution that was made using estimates of cognate replacement for 200 concepts on an Indo-European language tree spanning 6–10 millennia to measure lexical evolution rates demonstrated that negative valence correlates with faster cognate replacement, even while controlling for frequency of use. Follow-up analyses showed that it is most robust for adjectives, does not consistently reach statistical significance for verbs, and never reaches significance for nouns (Jackson et al. 2023).

Socio-demographic pressures are also known to lead to the emergence of new languages, the investigation of which can shed light on the process of language evolution. Among the newest languages of the world, Afrikaans is the youngest nationally recognized language, whose origin is tied to the establishment of Cape Colony in the Cape of Good Hope on the land of indigenous Khoekhoen people in the mid-16th century. The starting point of the Cape Colony is the landing of Jan van Riebeeck with three vessels to the Cape on 6th April 1652, which can also be thought of as the starting point of the Afrikaans language (De Villiers 2012). The language developed through to the 18th century, becoming the language with the widest geographical, demographic, and racial distribution of all official languages of South Africa (Webb 2003), and the debate on its origin was live and ongoing until the 20th century, mainly due the clash of political and ideological views that it instigated. Certain scholars at the time denied any indigenous influences on the language, while others insisted that the language was as much creolized as it was a product of West-Germanic sources. It has its roots in 17th-century Dutch but has been influenced by English, French, and German (Hamans 2021), with traces of, amongst others, Malay and Portuguese (Conradie and Groenewald 2014), and influenced by the pidgin talk of the indigenous Khoi and the San (Hamans 2021).

While not as widely recognized as Afrikaans, Light Warlpiri would constitute the newest example of an emerging language. This language was discovered and documented by Carmel O’Shannessy (2005) and is thought to have originated sometime at the end of the previous century. It was spoken in the Warlpiri community of Lajamanu, in the Northern Territory of Australia, by children and young adults who are now mostly approximately 40 years of age (O’Shannessy and Brown 2021). The language systematically combines elements of Warlpiri (a Pama-Nyungan language), Kriol (an English-based creole), and English, and was derived from the code-switched speech of parents to children following a particular pattern, where a Kriol pronoun and verb were inserted into a Warlpiri string, as part of a baby talk register (O’Shannessy 2012). A new language emerged when young children internalized this pattern of speech as a single system, distinct from Warlpiri (O’Shannessy 2020). In its formation, a speech pattern was further innovated in the verbal auxiliary system, namely, the =m “NONFUTURE” suffix (O’Shannessy 2013). Light Warlpiri has enough systematic evidence to distinguish it as a separate language, for which its precursors act as lexifiers (O’Shannessy 2005), and is nowadays the language of everyday interaction of the adult generation in the Lajamanu community.

To conclude, most typological studies suggest that linguistic diversity may be affected by several demographical and sociolinguistic factors. Social contexts can influence the way language is acquired and used, leading to linguistic structures that are specific to certain groups. Over time, this can result in variations in language usage that are reflected in typological patterns. This shows that language is not just a set of fixed rules and structures but rather a dynamic and adaptive skill that is shaped by the social context in which it is used. This ability to adapt to a social environment is a key feature of a language, and it helps to explain how language has evolved to meet the changing needs and contexts of different communities.

### 3.3. Technological Adaptations

Technology has drastically affected language and given rise to what is now commonly referred to as “text-speak” (Al-Sharqi and Abbasi 2020). One of the biggest changes technology brought about is the speed of communication. The human brain is forced to process an unending stream of linguistic input and respond to it immediately. Christiansen and Charter called this the “Now-or-Never bottleneck,” which describes the immediacy with which the brain must compress and recode linguistic input (Christiansen and Chater 2016). This bottleneck acts as a strong selection pressure against words and grammatical construction parsing, which, in real-time, is nearly impossible, especially when pressure is being put on the written language to communicate subtle nuances of face-to-face communication. The multi-faceted pressure inevitably influences the language that now has to undergo significant adaptive processes in order to fit the requirements of modern times, turning into what some deem “a natural experiment in the development of written communication” (Varnhagen et al. 2010). Some of the ways that this adaptation manifests in the language are novel conventions of online communication, including acronyms, the modified use of typographic marks, and the use of emojis (Lupyan and Dale 2016).

Emojis and emoticons are a group of symbolic combinations or pictures that are characteristic of online communication. An emoji (“☹”) is a graphic symbol that represents a wide variety of different things, ranging from complex facial expressions to concepts and ideas. It is thought to have developed from emoticons, i.e., representations of facial expressions usually comprised of various combinations of keyboard characters (“:)”). These symbols usually augment a message with non-verbal elements (Novak et al. 2015).

Due to their growing popularity, emojis are used not only in online communications but are becoming integrated into an increasingly wider variety of contexts. Specifically, research is conducted to understand how emoji-enriched interfaces affect performance in the classroom (Aliannejadi et al. 2021), marketing and advertisement (Lee et al. 2021), and even their implications in law (Goldman 2018).

Emojis (or “smileys”) are a unique phenomenon in terms of their nature and diverse functions in communication. On the one hand, emojis produce effects that are functionally similar to the response observed for facial expressions of emotion in face-to-face communication. They seem to affect the perceived emotional intensity of a message and accentuate its perceived valence by acting as nonverbal cues in digital communication (Erle et al. 2021). On the other hand, emojis are closely connected with words. It was shown that the time course of semantic congruency effects on eye movements for emojis is similar to effects that were previously shown for words (Barach et al. 2021). In the online public context, emojis alter the lexical diversity of text, which may point to a compensatory relationship between emojis and words in communication (Feldman et al. 2021). Additionally, there is a link between emojis and gestures, with emojis denoting objects and activities interacting with logical operators in a text in a similar way as gestures do with speech (Pierini 2021).

Lupyan and Dale argued that the divergence between conventional written languages (as well as online written communication) differs in many ways from the divergence between conventional spoken languages, for example, Dutch and Afrikaans. Both of these phenomena represent how languages (or language registers) adapt to the environments in which they are being used (Lupyan and Dale 2016). Similar to Afrikaans and Dutch, it is feasible to assume that the written form of language diverges from the spoken form as an adaptation to this new environment, and the online form combines the features of the two forms into something that linguist John McWhorter described as a pure “linguistic miracle happening right under our noses” (McWhorter 2013).

Shortcuts are one of the most prevalent features of the new “netspeak” (Varnhagen et al. 2010). Common shortcuts in the context of instant messaging and online communication include abbreviations (prof—professor), initialisms and acronyms (LOL—laughing out loud, ASAP—as soon as possible), and logograms or “alphanumeronyms” (CUL8R—see you later).

One of the explanations of this process posits that users intuitively ignore uneconomical language rules and strive for cost-effectiveness, increasing the efficiency of the language orthography (Lančarič 2016) and enriching it with new words and phrases that express complex feelings, emotions, or reactions (for example “wowzy” usually stands for extreme amazement or awe of a situation, thing, person, or place). Interestingly, some of the recently introduced units started to undergo a process of pragmaticalization, a subclass of grammaticalization, which possesses many similar features of grammaticalization processes but is distinguished from other subtypes by specific functions, domains, and syntactic integration (Diewald 2011).

Pragmaticalized units may partially or completely lose their semantic meaning and move into a new pragmatic domain of function and meaning. In this sense, online communication is not only enriched by the spoken form of language with its abundance of discourse (or pragmatic) markers but gives rise to new netspeak-specific pragmatic features that slowly pave their way back into spoken language, making this interaction bidirectional and mutually enriching. This is only one of many aspects that is indicative of the emergence of a unique new hybrid register that fuses the full range of variants from the language use, namely, written, spoken, formal, informal, and vernacular variants (Tagliamonte and Denis 2008).

One particular field able to inform the shortcut trend is quantitative linguistics, one of the aims of which is the development of statistical laws about language usage. Such laws can tell us a lot about speech and language efficiency principles, the most established among them being Zipf’s law of word frequency, which quantifies the frequency of occurrence of words, demonstrating that there is no unarbitrary way to distinguish between rare and common words (Zipf 1949). This law is also rather common in complex systems where discrete units self-organize into groups or types (Corral et al. 2019). Zipf’s law of brevity is sufficiently easier to observe through personal experience, stating that more frequent words tend to be shorter, and rarer words tend to be longer (Bentz and Ferrer-i-Cancho 2016). A functional explanation for this law suggested by Zipf is the law of least effort, stating that it is human nature to want the greatest outcome at the least amount of work. Closely related to it is the Menzerath–Altmann law, which postulates that the size of the constituents (e.g., phonemes) of a construction (e.g., morpheme) decreases with the increasing size of the construction (Altmann 1980). These two laws suggest that in human vocal communication, the maximization of coding efficiency and minimization of code length act as selective pressures to compress the elements supporting information (Favaro et al. 2020). In addition to spoken language, there is evidence that laws of brevity hold in most writing systems (in a sample of 1262 texts and 986 different languages, see Bentz and Ferrer-i-Cancho 2016). However, there are exceptions, as with figurative signals, the frequency of which is shown to be positively correlated with complexity (Miton and Morin 2019). The suggestion that these laws might extend beyond human language is substantivized by recent studies into vocal sequences of non-human primates (Semple et al. 2010) and penguins (Favaro et al. 2020), while some studies challenge that notion (Bezerra et al. 2011).

Hence, technology has had a significant impact on language in recent years, giving rise to new forms of communication and new ways of using language. One of the most notable changes brought about by technology is the emergence of “text-speak,” which is a form of language that is characterized by the use of shorthand, abbreviations, and symbols in text-based communication. In this way, digital communication can be seen as a distinct form of language with unique features and conventions that are specific to the digital environment. It is not a replacement for spoken or written language, but rather, it is an additional way of communicating and conveying meaning, which is a skill that emerged in humans as a response to a new and all-encompassing technological environment with its constraints and possibilities.

## 4. Conclusions

The questions of the origin and evolution of language, apart from gaining new evidence for their resolution through the use of novel linguistic and psycholinguistic methods and interdisciplinary inquiries, also changed in their nature. Modern language research strays away from portraying human language as a unique anthropoid phenomenon, the expression of which has not been modified by selection pressure, instead viewing it through the lens of ongoing human evolution and strict adherence to communicative goals. In this light, language can be viewed as a toolbox, the contents of which change in accordance with the needs of the human species, which is the statement that we attempted to demonstrate through the review of the literature on language evolution and adaptation.

Manifold examples of languages adapting to and reflecting different aspects of the environment illustrate that linguistic diversification is not simply an accumulation of random changes over time. The most recent example of strong selective pressure affecting languages all over the world is the integration of instant communication technologies. This new phenomenon allows us to witness language modification in real time and better understand the underlying processes. It can already be confidently stated that human language is undergoing one of its most massive changes at this very moment while following the essential principles that governed its existence and development before the onset of the digital age.

Multimodal and usage-based emergence theories, in addition to more robust correlation and causation links, provide a framework that is apt to incorporate the majority of scientific knowledge about language. Modern neural correlate models of language processing further serve to illustrate the interconnectedness of language to other domains of cognition. Further steps in that direction of inquiry may only serve to elucidate how thoroughly integrated language is with all other types of human behavior.

We propose that the study of languages should not be confined to properties of particular languages and language in general but should incorporate a wider array of contributing factors that inevitably shape the way different species, including humans, communicate. This all-encompassing approach will provide more insights into the nature, structure, and functions of language in diverse environments and demographic contexts, as well as help to explain the way human communication adapts to and transforms in response to the pressures put forward by technological breakthroughs and societal transformations, along with the alterations in our species’ ecological niche in the Anthropocene era.

## Data Availability

Not applicable.

## References

[B1-jintelligence-11-00061] Alemi Razieh, Batouli Seyed Amir Hossein, Behzad Ebrahim, Ebrahimpoor Mitra, Oghabian Mohammad Ali (2018). Not single brain areas but a network is involved in language: Applications in presurgical planning. Clinical Neurology and Neurosurgery.

[B2-jintelligence-11-00061] Aliannejadi Mohammad, Landoni Monica, Huibers Theo, Murgia Emiliana, Pera Maria Soledad (2021). Children’s Perspective on How Emojis Help Them to Recognise Relevant Results: Do Actions Speak Louder Than Words?. Paper presented at 2021 Conference on Human Information Interaction and Retrieval.

[B3-jintelligence-11-00061] Al-Sharqi Laila, Abbasi Irum Saeed (2020). The Influence of Technology on English Language and Literature. English Language Teaching.

[B4-jintelligence-11-00061] Altmann Gabriel (1980). Prolegomena to Menzerath’s law. Glottometrika.

[B5-jintelligence-11-00061] Anthropocene Working Group (2019). What Is the Anthropocene?—Current Definition and Status. http://quaternary.stratigraphy.org/working-groups/anthropocene/.

[B6-jintelligence-11-00061] Arbib Michael A. (2012). How the Brain Got Language: The Mirror System Hypothesis.

[B7-jintelligence-11-00061] Ardila Alfredo, Bernal Byron, Rosselli Monica (2016). How Localized are Language Brain Areas? A Review of Brodmann Areas Involvement in Oral Language. Archives of Clinical Neuropsychology.

[B8-jintelligence-11-00061] Ardila Alfredo (2011). There are two different language systems in the brain. Journal of Behavioral and Brain Science.

[B9-jintelligence-11-00061] Ardila Alfredo (2012). Interaction between lexical and grammatical language systems in the brain. Physics of Life Reviews.

[B10-jintelligence-11-00061] Atkinson Quentin D. (2011). Phonemic Diversity Supports a Serial Founder Effect Model of Language Expansion from Africa. Science.

[B11-jintelligence-11-00061] Baldo Juliana V., Dronkers Nina F. (2007). Neural correlates of arithmetic and language comprehension: A common substrate?. Neuropsychologia.

[B12-jintelligence-11-00061] Barach Elisa, Feldman Laurie Beth, Sheridan Heather (2021). Are emojis processed like words? Eye movements reveal the time course of semantic processing for emojified text. Psychonomic Bulletin & Review.

[B13-jintelligence-11-00061] Barbieri Chiara, Blasi Damián E., Arango-Isaza Epifanía, Sotiropoulos Alexandros G., Hammarström Harald, Wichmann Søren, Greenhill Simon J., Gray Russell D., Forkel Robert, Bickel Balthasar (2022). A global analysis of matches and mismatches between human genetic and linguistic histories. Proceedings of the National Academy of Sciences of the United States of America.

[B14-jintelligence-11-00061] Bastiaansen Marcel, Hagoort Peter, Neuper Christa, Klimesch Wolfgang (2006). Oscillatory neuronal dynamics during language comprehension. Progress in Brain Research.

[B15-jintelligence-11-00061] Bentz Christian, Ferrer-i-Cancho Ramon (2016). Zipf’s Law of Abbreviation as a Language Universal.

[B16-jintelligence-11-00061] Bentz Christian, Dediu Dan, Verkerk Annemarie, Jäger Gerhard (2018). The evolution of language families is shaped by the environment beyond neutral drift. Nature Human Behaviour.

[B17-jintelligence-11-00061] Bezerra Bruna M., Souto Antonio S., Radford Andrew N., Jones Gareth (2011). Brevity Is Not Always a Virtue in Primate Communication. Biology Letters.

[B18-jintelligence-11-00061] Boeckx Cedric (2021). Reflections on language evolution. Language Science Press.

[B19-jintelligence-11-00061] Bolhuis Johan J., Tattersall Ian, Chomsky Noam, Berwick Robert C. (2014). How Could Language Have Evolved?. PLoS Biology.

[B20-jintelligence-11-00061] Bouckaert Remco R., Lemey Philippe, Dunn Michael D., Greenhill Simon J., Alekseyenko Alexander V., Drummond Alexei J., Gray Russell D., Suchard Marc A., Atkinson Quentin D. (2012). Mapping the origins and expansion of the Indo-European language family. Science.

[B21-jintelligence-11-00061] Broca Paul (1861). Remarks on the seat of the faculty of articulated language, following an observation of aphemia (loss of speech). Bulletin de La Société Anatomique.

[B22-jintelligence-11-00061] Bromham Lindell, Hua Xia, Fitzpatrick Thomas G., Greenhill Simon J. (2015). Rate of language evolution is affected by population size. Proceedings of the National Academy of Sciences of the United States of America.

[B23-jintelligence-11-00061] Brown Angela M., Lindsey Delvin T. (2004). Color and language: Worldwide distribution of Daltonism and distinct words for “blue”. Visual Neuroscience.

[B24-jintelligence-11-00061] Busnel Rene-Guy, Classe André (1976). Whistled Languages.

[B25-jintelligence-11-00061] Chomsky Noam (1988). Language and Problems of Knowledge: The Managua Lectures.

[B26-jintelligence-11-00061] Christiansen Morten H., Chater Nick (2016). The Now-or-Never bottleneck: A fundamental constraint on language. Behavioral and Brain Sciences.

[B27-jintelligence-11-00061] Conradie Jac, Groenewald Gerald, Carstens W. A. M., Bosman N. (2014). Die ontstaan en vestiging van Afrikaans.

[B28-jintelligence-11-00061] Corballis Michael C. (2011). The Origins of Language in Manual Gestures.

[B29-jintelligence-11-00061] Corral Álvaro, Serra Isabel, Ferrer-i-Cancho Ramon (2019). Distinct flavors of Zipf’s law and its maximum likelihood fitting: Rank-size and size-distribution representations. ArXiv: Data Analysis, Statistics and Probability.

[B30-jintelligence-11-00061] Creanza Nicole, Ruhlen Merritt, Pemberton Trevor J., Rosenberg Noah A., Feldman Marcus W., Ramachandran Sohini (2015). A comparison of worldwide phonemic and genetic variation in human populations. Proceedings of the National Academy of Sciences of the United States of America.

[B31-jintelligence-11-00061] Dale Rick, Lupyan Gary (2012). Understanding the origins of morphological diversity: The linguistic niche hypothesis. Advances in Complex Systems.

[B32-jintelligence-11-00061] Darwin Charles (1860). On the Origin of Species by Means of Natural Selection: Or the Preservation of the Favoured Races in the Struggle for Life.

[B33-jintelligence-11-00061] Darwin Charles (1871). The Descent of Man, and Selection in Relation to Sex, Vol. 1.

[B34-jintelligence-11-00061] De Villiers Johan, Pretorius Fransjohan (2012). Die Nederlandse era aan die Kaap, 1652–1806. Geskiedenis van Suid-Afrika. Van Voortye Tot Vandag Bl.

[B35-jintelligence-11-00061] Dediu Dan, Boer Bart de (2016). Language evolution needs its own journal. Journal of Language Evolution.

[B36-jintelligence-11-00061] Dediu Dan, Janssen Rick, Moisik Scott R. (2017). Language is not isolated from its wider environment: Vocal tract influences on the evolution of speech and language. Language & Communication.

[B37-jintelligence-11-00061] Derrida Jacques (1976). Of Grammatology, Trans. Gayatri Chakravorty Spivak.

[B38-jintelligence-11-00061] Diewald Gabriele (2011). Pragmaticalization (Defined) as Grammaticalization of Discourse Functions.

[B39-jintelligence-11-00061] Donohue Mark, Denham Tim (2010). Farming and Language in Island Southeast Asia Reframing Austronesian History. Current Anthropology.

[B40-jintelligence-11-00061] Dronkers Nina F. (1996). A new brain region for coordinating speech articulation. Nature.

[B41-jintelligence-11-00061] Dunbar Robin I. M., Schmitt Alain, Atzwanger Klaus, Grammer Karl, Schäfer Katrin (1997). Groups, Gossip, and the Evolution of Language. New Aspects of Human Ethology.

[B42-jintelligence-11-00061] Erle Thorsten M., Schmid Karoline, Goslar Simon H., Martin Jared D. (2021). Emojis as social information in digital communication. Emotion.

[B43-jintelligence-11-00061] Everett Caleb, Blasi Damián E., Roberts Seán G. (2015). Climate, vocal folds, and tonal languages: Connecting the physiological and geographic dots. Proceedings of the National Academy of Sciences of the United States of America.

[B44-jintelligence-11-00061] Everett Caleb (2017). Languages in Drier Climates Use Fewer Vowels. Frontiers in Psychology.

[B45-jintelligence-11-00061] Favaro Livio, Gamba Marco, Cresta Eleonora, Fumagalli Elena, Bandoli Francesca, Pilenga Cristina, Isaja Valentina, Mathevon Nicolas, Reby David (2020). Do penguins’ vocal sequences conform to linguistic laws?. Biology Letters.

[B46-jintelligence-11-00061] Feldman Laurie, Barach Eliza, Srinivasan Vidhushini, Shaikh Samira (2021). Emojis and Words Work Together in the Service of Communication.

[B47-jintelligence-11-00061] Fitch William T. (2010). The Evolution of Language.

[B48-jintelligence-11-00061] Formigari Lia, Emanuele B. (2013). L’origine del linguaggio. Ricognizioni storiche e valenze epistemologiche. Sull’origine del Linguaggio e delle lingue storico-naturali. Un confronto tra linguisti e non linguisti.

[B49-jintelligence-11-00061] Goldman Eric (2018). Emojis and the Law. Washington Law Review.

[B50-jintelligence-11-00061] Goody Jack, Watt Ian (1963). The Consequences of Literacy. Comparative Studies in Society and History.

[B51-jintelligence-11-00061] Goody Jack (1986). *The Logic of Writing and the Organization of Society*, 1st ed. Cambridge: Cambridge University Press.

[B52-jintelligence-11-00061] Graffi Giorgio (2019). Origin of language and origin of languages. Evolutionary Linguistic Theory.

[B53-jintelligence-11-00061] Gray Russell D., Drummond Alexei J., Greenhill Simon J. (2009). Language Phylogenies Reveal Expansion Pulses and Pauses in Pacific Settlement. Science.

[B54-jintelligence-11-00061] Gray Russell D., Atkinson Quentin D. (2003). Language-tree divergence times support the Anatolian theory of Indo-European origin. Nature.

[B55-jintelligence-11-00061] Grigorenko Elena, Preiss David D., Kaufman James C., Singer Marcos (2023). The Never-Ending Innovativeness of the Wise Man. Innovation, Creativity and Change Across Cultures.

[B56-jintelligence-11-00061] Hagoort Peter (2005). On Broca, brain, and binding: A new framework. Trends in Cognitive Sciences.

[B57-jintelligence-11-00061] Hamans Camiel (2021). Afrikaans: A language where ideology and linguistics meet. Scripta Neophilologica Posnaniensia.

[B58-jintelligence-11-00061] Hauser Mark D., Chomsky Noam, Fitch William T. (2002). The faculty of language: What is it, who has it, and how did it evolve?. Science.

[B59-jintelligence-11-00061] Jackson Joshua Conrad, Lindquist Kristen, Drabble Ryan, Atkinson Quentin, Watts Joseph (2023). Valence-dependent mutation in lexical evolution. Nature Human Behaviour.

[B60-jintelligence-11-00061] Winters James, Kirby Simon, Smith Kenny (2015). Languages adapt to their contextual niche. Language and Cognition.

[B61-jintelligence-11-00061] Jordan Kirsten, Heinze H. -J., Lutz Kai, Kanowski Martin, Jäncke Lutz (2001). Cortical activations during the mental rotation of different visual objects. Neuroimage.

[B62-jintelligence-11-00061] Koplenig Alexander (2019). Language structure is influenced by the number of speakers but seemingly not by the proportion of non-native speakers. Royal Society Open Science.

[B63-jintelligence-11-00061] Labov William (1991). Sociolinguistic Patterns.

[B64-jintelligence-11-00061] Lančarič Daniel (2016). Sentential Acronyms in Informal Online Communication. Reviewed Conference Proceedings from an International Scientific Conference. https://faj.euba.sk/www_write/files/veda-vyskum/konferencie/zborniky/Cudzie_jazyky_v_premenach_casu_7_2016.pdf#page=10.

[B65-jintelligence-11-00061] Lee Jungwoo, Kim Cheong, Lee Kun Chang (2021). Investigating the Negative Effects of Emojis in Facebook Sponsored Ads for Establishing Sustainable Marketing in Social Media. Sustainability.

[B66-jintelligence-11-00061] Levinson Stephen C., Holler Judith (2014). The origin of human multi-modal communication. Philosophical Transactions of the Royal Society B.

[B67-jintelligence-11-00061] Lewis Molly, Frank Michael C. (2016). Linguistic niches emerge from pressures at multiple timescales. Cognitive Science.

[B68-jintelligence-11-00061] Lewis Paul M. (2009). Ethnologue: Languages of the World.

[B69-jintelligence-11-00061] Lindsey Delvin T., Brown Angela M. (2002). Color Naming and the Phototoxic Effects of Sunlight on the Eye. Psychological Science.

[B70-jintelligence-11-00061] Linell Per (2004). The Written Language Bias in Linguistics: Its Nature, Origins and Transformations.

[B71-jintelligence-11-00061] Lock Andy, Gers Matt (2012). The cultural evolution of written language and its effects: A Darwinian process from prehistory to the modern day. Writing: A Mosaic of New Perspectives.

[B72-jintelligence-11-00061] Lupyan Gary, Dale Rick (2010). Language Structure Is Partly Determined by Social Structure. PLoS ONE.

[B73-jintelligence-11-00061] Lupyan Gary, Dale Rick (2016). Why Are There Different Languages? The Role of Adaptation in Linguistic Diversity. Trends in Cognitive Sciences.

[B74-jintelligence-11-00061] Mace Ruth, Holden Clare (2005). A phylogenetic approach to cultural evolution. Trends in Ecology and Evolution.

[B75-jintelligence-11-00061] MacNeilage Peter F. (2008). The Origin of Speech.

[B76-jintelligence-11-00061] Maddieson I. (2018). Language Adapts to Environment: Sonority and Temperature. Frontiers in Communication.

[B77-jintelligence-11-00061] Maddieson Ian, Coupé Christophe (2015). Human spoken language diversity and the acoustic adaptation hypothesis. The Journal of the Acoustical Society of America.

[B78-jintelligence-11-00061] Maess Burkhard, Koelsch Stefan, Gunter Thomas C, Friederici Angela D (2001). Musical syntax is processed in Broca’s area: An MEG study. Nature Neuroscience.

[B79-jintelligence-11-00061] McLuhan Marshall (1962). The Gutenberg Galaxy: The Making of Typographic Man.

[B80-jintelligence-11-00061] McWhorter John (2013). John McWhorter: Txtng is killing language. JK!!!|TED Talk. https://www.ted.com/talks/john_mcwhorter_txtng_is_killing_language_jk.

[B81-jintelligence-11-00061] Mendívil-Giró José-Luis (2018). Why Don’t Languages Adapt to Their Environment?. Frontiers in Communication.

[B82-jintelligence-11-00061] Meyer Julien, Magnasco Marcelo O., Reiss Diana (2021). The Relevance of Human Whistled Languages for the Analysis and Decoding of Dolphin Communication. Frontiers in Psychology.

[B83-jintelligence-11-00061] Meyer Julien (2015). Whistled Languages.

[B84-jintelligence-11-00061] Meyer Julien (2018). Revitalization of Whistled Languages. The Routledge Handbook of Language Revitalization.

[B85-jintelligence-11-00061] Miton Helen, Morin Olivier (2019). When iconicity stands in the way of abbreviation: No Zipfian effect for figurative signals. PLoS ONE.

[B86-jintelligence-11-00061] Nettle Daniel, Romaine Suzanne (2000). Vanishing Voices: The Extinction of the World’s Languages.

[B87-jintelligence-11-00061] Nölle Jonas, Fusaroli Riccardo, Mills Gregory J., Tylén Kristian (2020a). Language as shaped by the environment: Linguistic construal in a collaborative spatial task. Palgrave Communications.

[B88-jintelligence-11-00061] Nölle Jonas, Hartmann Stefan, Tinits Peeter (2020b). Language Evolution Research in the Year 2020: A Survey of New Directions. Language Dynamics and Change.

[B89-jintelligence-11-00061] O’Shannessy Carmel, Brown Connor (2021). Reflexive and Reciprocal Encoding in the Australian Mixed Language, Light Warlpiri. Langages.

[B90-jintelligence-11-00061] O’Shannessy Carmel (2005). Light Warlpiri: A new language. Australian Journal of Linguistics.

[B91-jintelligence-11-00061] O’Shannessy Carmel (2012). The role of codeswitched input to children in the origin of a new mixed language. Linguistics.

[B92-jintelligence-11-00061] O’Shannessy Carmel (2013). The role of multiple sources in the formation of an innovative auxiliary category in Light Warlpiri, a new Australian mixed language. Language.

[B93-jintelligence-11-00061] O’Shannessy Carmel (2020). How ordinary child language acquisition processes can lead to the unusual outcome of a mixed language. International Journal of Bilingualism.

[B94-jintelligence-11-00061] Obler Loraine K., Gjerlow Kris (1999). Language and the Brain.

[B95-jintelligence-11-00061] Olson David R. (2005). Technology and intelligence in a literate society. Intelligence and Technology.

[B96-jintelligence-11-00061] Olson David R. (2012). Language, literacy and mind: The literacy hypothesis. Writing: A Mosaic of New Perspectives.

[B97-jintelligence-11-00061] Open Science Collaboration (2015). Estimating the reproducibility of psychological science. Science.

[B98-jintelligence-11-00061] Passmore Sam, Wood Anna L. C., Barbieri Chiara, Barbieri Chiara, Shilton Dor, Daikoku Hideo, Atkinson Quentin, Savage Patrick E. (2022). Global relationships between musical, linguistic, and genetic diversity. PsyArXiv.

[B99-jintelligence-11-00061] Pearl Judea (2000). Causality: Models, Reasoning, and Inference.

[B100-jintelligence-11-00061] Pérez-Losada Joaquim, Fort Joaquim (2018). A serial founder effect model of phonemic diversity based on phonemic loss in low-density populations. PLoS ONE.

[B101-jintelligence-11-00061] Perlman Marcus (2017). Debunking two myths against vocal origins of language: Language is iconic and multimodal to the core. Interaction Studies. Social Behaviour and Communication in Biological and Artificial Systems.

[B102-jintelligence-11-00061] Novak Petra Kralj, Smailović Jasmina, Sluban Borut, Mozetič Igor (2015). Sentiment of Emojis. PLoS ONE.

[B103-jintelligence-11-00061] Pierini Francesco (2021). Emojis and gestures: A new typology. Proceedings of Sinn Und Bedeutung.

[B104-jintelligence-11-00061] Pinker Steven, Bloom Paul (1990). Natural language and natural selection. Behavioral and Brain Sciences.

[B105-jintelligence-11-00061] Pleyer Michael, Hartmann Stefen (2019). Constructing a Consensus on Language Evolution? Convergences and Differences Between Biolinguistic and Usage-Based Approaches. Frontiers in Psychology.

[B106-jintelligence-11-00061] Preiss David D., Sternberg Robert J., Preiss David D. (2022). Human Intelligence in the Time of the Anthropocene. Intelligence in Context: The Cultural and Historical Foundations of Human Intelligence.

[B107-jintelligence-11-00061] Preiss David D., Sternberg Robert J. (2006). Effects of technology on verbal and visual-spatial abilities. International Journal of Cognitive Technology.

[B108-jintelligence-11-00061] Prugnolle Franck, Manica Andrea, Balloux Francois (2005). Geography predicts neutral genetic diversity of human populations. Current Biology.

[B109-jintelligence-11-00061] Ramachandran Sohini, Deshpande Omkar, Roseman Charles C., Rosenberg Noah A., Feldman Marcus W., Cavalli-Sforza Luigi Luca (2005). Support from the relationship of genetic and geographic distance in human populations for a serial founder effect originating in Africa. Proceedings of the National Academy of Sciences of the United States of America.

[B110-jintelligence-11-00061] Rizzolatti Giacomo, Arbib Michael A. (1998). Language within our grasp. Trends in Neurosciences.

[B111-jintelligence-11-00061] Roberts Sean G. (2018). Robust, Causal, and Incremental Approaches to Investigating Linguistic Adaptation. Frontiers in Psychology.

[B112-jintelligence-11-00061] Roberts Seán G., Killin Anton, Deb Angarika, Sheard Catherine, Greenhill Simon J., Sinnemäki Kaius, Segovia-Martín José, Nölle Jonas, Berdicevskis Aleksandrs, Humphreys-Balkwill Archie (2020). CHIELD: The causal hypotheses in evolutionary linguistics database. Journal of Language Evolution.

[B113-jintelligence-11-00061] Roberts Sean, Winters James (2012). Social Structure and Language Structure: The New Nomothetic Approach. Psychology of Language and Communication.

[B114-jintelligence-11-00061] Robinson Elizabeth, Goelman Hillel, Olson David R. (1983). Children’s understanding of the relation between expressions (what was said) and intentions (what was meant). British Journal of Developmental Psychology.

[B115-jintelligence-11-00061] Tagliamonte Sali A., Denis Derek (2008). Linguistic ruin? Lol! Instant messaging and teen language. American Speech.

[B116-jintelligence-11-00061] Saussure Ferdinand de, Bally Charles, Sechehaye Albert, Riedlinger Albert (1986). Course in General Linguistics.

[B117-jintelligence-11-00061] Schmandt-Besserat Denise (2012). Tokens as precursors of writing. Writing: A Mosaic of New Perspectives.

[B118-jintelligence-11-00061] Semple Stuart, Hsu Minna J., Agoramoorthy Govindasamy (2010). Efficiency of coding in macaque vocal communication. Biology Letters.

[B119-jintelligence-11-00061] Tomasello Michael, Bavin Edith L. (2009). The usage-based theory of language acquisition. The Cambridge Handbook of Child Language.

[B120-jintelligence-11-00061] Varnhagen Connie K., McFall G. Peggy, Pugh Nicole, Routledge Lisa, Sumida-MacDonald Heather, Kwong Trudy E. (2010). lol: New language and spelling in instant messaging. Reading and Writing.

[B121-jintelligence-11-00061] Webb Vic (2003). Language Policy in Post-Apartheid South Africa. Medium of Instruction Policies.

[B122-jintelligence-11-00061] Wernicke Carl, Eling Paul (1994). A Psychological Study on an Anatomical Basis: The Aphasia Symptom-Complex. Reader in the History of Aphasia: From Franz Gall to Norman Geschwind.

[B123-jintelligence-11-00061] Zipf George Kingsley (1949). Human Behavior and the Principle of Least Effort.

[B124-jintelligence-11-00061] Zlatev Jordan, Wacewicz Sławomir, Zywiczynski Przemyslaw, Weijer Joost van de (2017). Multimodal-first or pantomime-first? Communicating events through pantomime with and without vocalization. Interaction Studies. Social Behaviour and Communication in Biological and Artificial Systems.

